# Cognitive Reframing of Intimate Partner Aggression: Social and Contextual Influences

**DOI:** 10.3390/ijerph15112464

**Published:** 2018-11-05

**Authors:** Wind Goodfriend, Ximena B. Arriaga

**Affiliations:** 1 Psychology Department, Buena Vista University, Storm Lake, IA 50588, USA; 2 Department of Psychological Sciences, Purdue University, West Lafayette, IN 47907, USA

**Keywords:** intimate partner aggression, perceptions of IPA or IPV, coping with IPA or IPV

## Abstract

Intimate partner aggression violates U.S. culturally-accepted standards regarding how partners should treat each other. Victims must reconcile the dissonance associated with being in what should be a loving and supportive relationship, while being in the same relationship that is personally and deeply harmful. To manage these clashing cognitions, victims consciously and unconsciously adopt perceptions to reframe their partner’s aggression, minimizing and reinterpreting the occurrence or impact of aggressive acts, and justifying remaining in their relationship. The paper examines the multiple and nested influences that shape such perceptions, including individual, partner, relationship, and cultural factors. Each type of influence is discussed by reviewing previous research and including accounts from women who had experienced aggression. Greater awareness of such perceptions may afford greater control in changing harmful relationship patterns.

## 1. Introduction

If beauty lies in the eyes of the beholder, so, too, does ugliness. Perceptions of others go beyond assessments of physical beauty or ugliness and include interpretations of others’ behaviors. What one person may view as ugly behavior, another may perceive differently. How do victims of intimate partner aggression (IPA) perceive their partner, the person who has perpetrated the aggression? Previous research has established that individual perceptions of IPA vary. How each individual perceives and copes with his or her experience may be influenced by a variety of factors. Social norms (e.g., [1]) and scripts regarding what is expected from intimate partners (e.g., [2,3]) are well established in most cultures. In modern-day western societies, relationship partners are expected to fall in love and then support and comfort each other through times of stress or pain. However, what happens when that stress or pain comes from a partner? The aim of this article is to propose a framework for examining various influences on perceptions of individuals when they experience intimate partner aggression from their current relationship partner.

While many past studies have referred to “IPV” (intimate partner violence), typically that term focuses on physical forms of aggression, such as kicking or punching [4]. We instead use the term IPA (intimate partner aggression) to include a wider variety of aggression, including verbal, emotional, psychological, physical, or sexual aggression [5]. Indeed, victims of psychological or emotional aggression develop long-term negative effects—such as low self-esteem, depression, and anxiety—that may outlast the effects of physical aggression [6,7,8]. In addition, while physical forms of aggression are relatively easier to classify as “abuse”, non-physical forms are more subjective and thus may be harder for victims to conceptualize and process.

We begin by discussing the importance of cognitive reframing in making sense of IPA experiences. Perceptions of IPA may best be understood by examining a variety of social and contextual influences, including personal, partner, relational, and societal influences. Previous work provides insight into the value in breaking down levels of influence in this way. We present an analysis of each level of influence on perceptions, including both relevant work by other researchers and original data from our own interviews with current victims of intimate partner aggression. The original data are in the form of qualitative excerpts from interviewees that illustrate cognitive reframing processes, such as minimization, reinterpretation, and justification.

### 1.1. The Importance of Cognitive Reframing

Because IPA goes against culturally-accepted behaviors for how partners should treat each other, victims may experience intense cognitive dissonance [9,10]. The idea of a loving partner is dissonant with the idea that one’s partner is aggressive and violent. To manage these clashing cognitions, victims may (consciously or unconsciously) engage in cognitive processes that minimize the apparent occurrence or impact of aggressive acts, reinterpret their perceptions of the perpetrator, and justify remaining in the relationship despite the aggression (e.g., [11,12]).

Cognitive reframing processes can temporarily help victims cope with being in IPA situations when they are not able to end their relationship, essentially by providing justifications for staying. Over time, if they become less dependent (e.g., they gain access to financial resources, new experiences diminish their perceptions of their relationship) or if the aggression escalates making it increasingly less deniable, victims may cease their perceptions that justify aggression. Thus, cognitive reframing is subjective, may be temporary, and is likely influenced by the individual’s current psychological and social environment.

We discuss the wide variety of social and contextual factors that may influence subjective perceptions and interpretations of partner aggression, and specifically examine four levels of factors that are at play within IPA couple dynamics: individual factors, partner factors, relationship factors, and cultural factors. These levels thus move from more proximal/personal to more distal/general in their immediate effects on individual perceptions.

### 1.2. Gaining a Comprehensive View: An Ecological Approach

Researchers are increasingly focusing on how victims of IPA manage perceptions of their partners and aggressive acts in the relationship. One notable example is a study in which adults from a community college were asked about specific behaviors their partner may have done during a conflict situation [13]. For highly committed participants, while they admitted that their partner had done things like “kicked me” or “beat me up”, they interpreted these actions as “just joking around.” Presumably, the same behaviors would have been identified as aggressive violence by either outside observers or by less committed partners. The motivation to interpret IPA as less serious or as a “joke” likely stems from partners who feel involuntarily dependent on the relationship and cope with the aggression by downplaying it.

The specific type of cognitive reframing could, theoretically, take on a wide variety of forms (i.e., “joking” is just one of many possible ways to adopt an alternative view of what is happening in a relationship). To get a comprehensive view, we take an ecological approach encompassing the many factors that influence victims’ perceptions. The ecological approach developed by Uri Bronfenbrenner—one of the most influential scholars in the study of human development—examines people, their immediate environments, and the larger social contexts in which people are embedded [14].

Bronfenbrenner’s early work on a bioecological model examined the various systems that exert an influence on individual perceptions and actions. Immediate and proximal sources may be nested within more distal sources, all of which simultaneously affect an individual at the center, much like considering various layers that constitute nested Russian dolls [14]. For example, an individual’s biology and psychology operate in an immediate environment (e.g., one’s place of work), creating a “microsystem”. The connections among microsystems (e.g., work plus home environment) comprise a “mesosystem”, and a third layer—the exosystem—may include larger contexts such as neighborhoods, mass media, and government agencies. The largest structure is the “macrosystem”, which refers to global, cultural, or sub-cultural systems. Bronfenbrenner eventually added the “chronosystem”, or changes over time, as well as genetic inheritance [15].

Later, Bronfenbrenner expanded his model by explaining levels that he labeled Person-Process-Context-Time (or PPCT; [16,17]). Here, “person” refers to individual characteristics (e.g., outward appearance, personality, intelligence), “process” is the interaction between someone and his or her environment, “context” is the nested systems from micro- to macrosystem described in the paragraph above, and “time” is the chronosystem. Bronfenbrenner’s bioecological model has been applied in a wide variety of settings, such as infant-mother bonding [14,18]; influences on child abuse [19], community violence and child maltreatment [20], adolescent sexual activity [21], and health promotion [22].

Bronfenbrenner’s model has been applied to examine interpersonal violence, including violence in military groups or towards individuals with disabilities, responses to sexual aggression, and intervention efforts (see, for example, [23,24,25,26]). Perhaps the most relevant analysis for the purposes of the current paper was a study of mental health outcomes among victims of sexual assault [27]. The analysis revealed that individual reactions to assault changed based on multiple ecological levels or contexts ranging from individual factors about the assault victim (e.g., demographics, education, and pre-existing mental health conditions) through factors about the specific assault incident (e.g., who was the perpetrator, was a weapon involved, were threats used) to larger cultural factors (e.g., belief in rape myths, cultural stereotypes about victims, and the presence or absence of community resources).

We follow the same type of structure in our own analysis of levels of influence regarding how current victims of intimate partner aggression perceive themselves, their partner, their relationship, and the aggression within a larger cultural/societal context. We briefly describe the method of obtaining examples that illustrate the model, and then detail the model in a later section.

### 1.3. Interview Data

To explore the phenomenon of cognitive reframing of IPA, we interviewed 27 English-speaking women who were at a local domestic violence shelter for women. All interviews occurred between September 2001 and April 2002, and the study was approved by the hosting institution’s Institutional Review Board. Each woman was paid $25, and interviews lasted between 60–90 min. To start, all of the women read a consent form that explained: “I will ask you questions about your intimate relationship, including how you feel about your relationship, what your interaction and disagreements are like, and how you and your partner respond during and after conflicts, including whether each of you use physical force.” They were told that they could skip any questions they did not want to answer and that the interviews would be tape-recorded. Interviews were structured such that all participants were asked the same questions, in the same order.

The interviews covered a series of questions regarding the women’s relationship status, demographics, and views about relationship aggression. For this article, one question in particular was important. The women were asked if either their current partner or a past partner had used aggression, and, if so, to think specifically about the “most severe” incident that had occurred. For that particular incident, they were asked: “What did you do to deal with the event? What did you do to cope with the event?”

Participants’ answers were then coded using the structure identified in the next section (four specific levels, including individual, partner, relationship, and cultural). Thus, the coding scheme was created in advance, and after the data were collected the coding procedure was conducted informally as a way to explore particular women’s experiences and perceptions. As we detail the model below, we utilize the women’s responses as examples to illustrate each level of influence. We slightly altered some of the women’s responses without changing the meaning (e.g., rewording, removing potentially identifying comments) to maintain their confidentiality.

## 2. Four Levels of Influence

**Figure 1:**
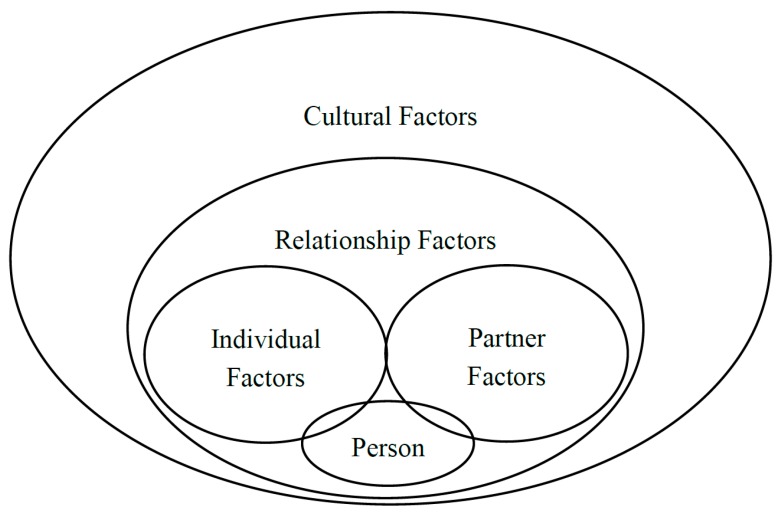
Levels of influence in perceptions of intimate partner aggression.

Figure 1 illustrates our model, with four levels of influence for perceptions of IPA. The person lies in the center, with individual factors and partner factors both affecting how that person thinks about relationship events, including aggression. Beyond that, relationship factors encompass both partners as they interact with each other. Finally, one’s culture affects social norms, beliefs, scripts, stereotypes, and general working models about relationships and the world.

### 2.1. Individual Influences and Self-Blame

At the most immediate and personal level for any given person are individual influences. These are factors tied directly to the person in terms of his or her life history, personality, intelligence, and so on. In the context of partner aggression, a common variable at this level is experience with violence or aggression in one’s family of origin. People who grow up in a violent or aggressive household are more likely to pass that aggression onto their own adult relationships, a cycle called intergenerational transmission (e.g., [28,29]). People who have been exposed to more aggression in the typical relationships surrounding them are more likely to indicate that they would tolerate aggression in their own relationships [30] (Study 1). Because of their own personal experiences earlier in life, these individuals may now perceive IPA as “normal”.

Biased perceptions of IPA can also be seen in people who have been the victim of aggression in their adult relationships. While most people categorize severe acts of aggression (such as physical violence or destroying one’s possessions) as being unacceptable, people who have personally experienced IPA have more extreme thresholds regarding the severity of aggressive acts that would warrant leaving a partner [30]. Personally experiencing IPA may lead to habituating and adjusting standards, and thus becoming more lenient toward future aggressive acts.

Some individuals begin to adjust their standards after a partner first becomes aggressive (see [30], Study 3). One woman we interviewed had gone through this very process. She stated, “Since it was my first week living with him, I was thinking, ‘Will it be more than just this? Is it going to be worse? Or is it just one time?’ What’s going through his head to make him do that?” Her confusion and dissonance are apparent as she attempts to understand what happened and categorize it, based on her understanding of what happens in relationships and what aggression looks like. This participant, later in the interview, suggested that her partner’s actions may have been due to alcohol and/or mental health concerns. She further stated, “He can go through three 30-packs [of alcohol] a day. And he’ll start at four in the morning. It’s bad.” This level of drinking is excessive, but she may have habituated to it and slowly changed her threshold of “bad” over time. The IPA certainly did not happen “just one time” as she originally wondered, but she stayed in the relationship anyway.

When people have experienced IPA throughout their lives from multiple different partners, one possible unfortunate perception that may arise is that the aggression is somehow their own fault [27]. Thus, self-blame may be considered an important individual-level influence on how specific incidents of IPA are perceived. Certainly, perpetrators of IPA will often blame their victims, stating that they have been “provoked” or that their victims are responsible for what is happening (e.g., the perpetrator is teaching them a “lesson”).

This individual self-blame came up in five out of our 27 interviews. The simplest and most straightforward comment from one participant was, “I am partly to blame for all this.” Another participant expanded the idea with these words: “I don’t know if you’ve ever had anybody who was really good about turning the tables on you and making it all look like it’s you. They’re always right.” She seemed to be aware of the manipulation from her partner that would cause her cognitive reframing in the form of self-blame. Another participant said, “Blame myself? I guess I took some responsibility… maybe I pushed his buttons. I never really learned to back down when someone is upset with me.”

A fourth participant noted that there was a specific cause of the IPA incident, but she took the blame on herself, despite the severity of the incident: “He got mad and kicked me in the stomach and I ended up going to the hospital. I guess I didn’t clean the house the right way; there was stuff all over the floor, my son was getting into stuff, and I wasn’t paying attention to him.” Finally, a particularly reflective participant offered the following interpretation:

“I just assumed, ‘Well, maybe I did do something wrong. I shouldn’t have let his friend come into the house and stand there asking me where my husband was.’ Maybe I should have seen his friend pull up and gone outside and said, ‘He’s not here.’ Maybe there was something else I could have done… because most of the things that happened with him occurred when he was drunk. There must be something that I’m doing wrong if he’s hitting me or beating me up…. I might have done something wrong. You know, nobody deserves to get treated that way, but I didn’t know back then… I was young. Maybe he’s right. Maybe there’s something that I’m doing wrong.”

This final quotation shows the individual level of self-blame and also previews partner influence. She perceived that she inevitably may have been the “cause” of the problem, but she also noted that her partner was drunk. Thus, there were two instigators in her mind: her own behavior and her husband’s state of drunkenness. This leads to the second level of influence, partner factors.

### 2.2. Partner Influences and “Uncontrollable Personality”

When a victim of IPA remains highly committed to the relationship or continues to feel closely connected with or attached to the perpetrator of that aggression, cognitive dissonance is particularly high. Many individuals may reinterpret aggression in order to reconcile positive feelings about their partner and negative feelings about the aggression they are experiencing. One way to reconcile these feelings is to perceive that the perpetrator is, him- or herself, a victim of a larger problem, such as a mental health issue or addiction. Within this reframing structure, a partner is not in control of the negative behaviors.

Many studies have examined the psychology of IPA perpetrators and have found evidence that, indeed, in many cases perpetrators have mental health issues. For example, clinical researchers have identified antisocial, narcissistic, or borderline personality disorders as risk factors for partner or family violence [31,32,33]. The manner in which victims of IPA may be blaming their partner’s mental health issues was summed up by one of our interview participants, who simply stated, “I knew my husband has an explosive personality.”

Holtzworth-Munroe and Stuart [34] argue that perpetrators can be classified using three characteristics: severity/frequency of aggression, how general the aggression is (within-family only or not), and degree of perpetrator pathology. In addition to a focus on personality disorders in perpetrators, hundreds of studies have found a link between aggressive behaviors (both within and outside of intimate relationships) and post-traumatic stress disorder (PTSD; [35,36,37,38]).

The link between IPA and PTSD may be particularly common in veterans [39,40,41,42,43]. This link was uncommon but nonetheless occurred among our interviewees. While only two participants specifically mentioned it, both used their partner’s veteran status as a justification to stay in the relationship and as a way to perceive that the aggression was not their partner’s fault. One participant noted, “It wasn’t that he wanted to hurt me…it was the problems he’s had as a result of the military and his first marriage.” Another provided this more detailed description:

“He wasn’t a violent person… he’s a disabled veteran… post-traumatic stress disorder. Things happened when he was having a flashback, or if he was drinking. It was mainly at night while he’s asleep… if I would move, it would startle him, and he didn’t know where he was. Sometimes he’d grab me and put his fist in my face and stop short of my nose. One time he choked me. He was yelling at me in a language I didn’t understand and choking me. Most of it really was verbal, emotional… a lot of manipulating, manipulation. Then he realized where he was and who I was… but he’d been drinking, so he didn’t remember it. I just spent the night in a corner, terrified.”

Unfortunately, PTSD is often comorbid with alcohol and drug use [44,45,46]. One of the most pervasive and ubiquitous findings within research on IPA is a correlation between aggressive incidents and substance use [47,48,49,50,51].

One study specifically investigated how the presence of alcohol changed people’s perceptions of IPA [52]. College students read vignettes describing increasingly aggressive scenarios between a dating couple in college. Half of the participants read scenarios in which alcohol was involved, and half did not. When the vignettes described psychological aggression or less severe forms of physical aggression, they saw the presence of alcohol as a mitigating circumstance and thus perceived the aggression as both more common and as less abusive. In short, they placed less blame on the perpetrator if he was drinking.

Using alcohol to downplay aggression came up frequently in our interviews, with four women explicitly mentioning it as the cause of a particularly violent incident in their own relationship. One woman said, “When he drinks too much… then, that’s what you watch out for, because he not in a stable mind.” Another noted, “I just don’t think he can control it. I just think he’s sick in the head or something… He drinks [alcohol] a lot, but I’ve seen him go a day without alcohol, and it’s just like terrible. Either way, he’s worse.”

A third interviewee reflected, “He had been drinking… he’s a hardcore alcoholic…. I didn’t realize it was that bad... I didn’t get it, really. I really couldn’t. And he couldn’t either, you know, he was sorry… he always blamed it on the liquor.” Finally, one participant combined the idea of substance abuse and PTSD when she stated fairly simply, “My husband also has a substance abuse problem, so that coupled with the PTSD is a very bad combination.”

### 2.3. Relationship Influences and “For Better or Worse”

To fully understand anything that happens within an intimate relationship, focusing on only one of the partners involved provides a myopic view. Thus, “dyadic” approaches to studying IPA attempt to incorporate aspects of both partners and of their couple dynamics [53]. One such dyadic model [54] first focuses on individual partner characteristics, then emphasizes ongoing social influences, peer influences, and developments over time [55]. These authors note that in longitudinal research, men’s IPA is relatively stable within a given relationship but changes significantly with different partners over time.

Thus, the dynamics of any given couple pairing may influence how partners act and the attributions each partner makes regarding the other’s behavior. In important work regarding typologies of abusers, Johnson [56,57] proposed that some couples exhibit volatile dynamics in which their conflicts escalate to violence; he called this “situational couple violence.” In an alternative typology model, Finkel [58] suggested that IPA is more likely in couples when certain triggers exist, such as when one partner tries to control the other or when one partner suspects the other of infidelity. Thus, beyond individual or partner influences on perceptions of IPA, an understanding of the relationship partners as a unit is also needed for a full view of why a victim of aggression might cognitively reframe those acts in certain ways.

A centrally important characteristic of each couple will be the extent that they rely on their relationship, as often is captured by their subjective statements about their commitment [59]. Having an aggressive partner poses a threat because it undermines one’s sense of well-being [60] and often is socially stigmatizing [30]. Despite these negative outcomes of IPA, in addition to many other harmful effects, high commitment or dependency may motivate individuals to cognitively reframe the aggression. When partners feel attached to each other, assume they will be together, have become complacent in their relationship or rely on each other day to day—emotionally or financially—they are more likely to protect their relationship against threats.

The state of relying on a partner—subjectively experienced as being committed—thus can influence the manner in which victims of IPA cognitively reframe aggression. The previous finding regarding a partner’s violence reframed as merely joking was unique to highly committed individuals [13]. Research on tolerance for aggression reveals that the exact same aggressive behavior is judged as more acceptable when it occurs in one’s own relationship than in a stranger’s relationship, especially when a person feels committed [30] (Study 3).

Over time, people who experience IPA victimization become increasingly tolerant and continue to justify staying in the relationship—but only if they are committed. As reviewed elsewhere [5], individuals who rely on their partner and feel committed: (1) place less blame on their aggressors [61], (2) make more excuses for their aggressors [62], and (3) are both more likely to stay in an aggressive relationship and return to their partners after temporarily leaving due to abuse [63,64].

One reason partners might be committed to each other is high levels of investments, or resources that are either already tied to the relationship or planned for the future; these resources would be lost if the relationship ended [64,65]. Investments may include time devoted to a relationship, children with a partner, sacrifices made to be together, and even future plans together. One of the women we interviewed was explicitly aware of how investments and commitment kept her in the relationship despite the aggression:

“Well, we were together for many years, and it was that we had so much invested in it… It’s not his fault, he’s a veteran, for better or for worse… and I didn’t want to take his son away from him. I wanted his son to have a father. So many years of marriage invested…. He couldn’t take care of himself because he’s ill, and I didn’t want to take my son and I didn’t want to be away from my son.”

Another reason why couple members might be dependent and feel committed is pressure from their family members to stay together, or pressure they put on themselves due to not wanting to let other people down [66]. One participant noted, “Sometimes with family—at least my family—you have to explain yourself so much, and, you know, they’re like … You need to have a husband provide (for you), and maybe it’s not as bad as you think.”

Similarly, two participants noted that they remained committed because one partner’s parents had also experienced IPA and had remained in the relationship. In one couple, it was the perpetrator’s mother: “His dad was abusive to his mom, and she stuck through it. He always brings that up, ‘my dad had problems, but my mother was strong and they got through it.’” Another woman remembered her own mother’s experience: “I never really thought about leaving. My mother stuck it out, so I figured I’d just stick it out and hopefully things would change.”

### 2.4. Cultural Influences and Redefining Violence

As shown in Figure 1, the most global and distal influence on perceptions of IPA are cultural factors. Culture can affect all of the levels of influence already reviewed (individual, partner, and relationship), but it can also reflect perceptions and acceptance of violence by people in general, beyond those individuals who themselves are experiencing aggression. One avenue in which culture affects perceptions is through people’s understanding of how common IPA is and whether IPA victims should put up with it. U.S. cultural norms from the 1950s may have emphasized the “for better or worse” expectations of marriage, whereas views of inter-partner abuse became less accepting during the 1990s [67].

In cultures in which patriarchal beliefs and/or sexism are prevalent or more ingrained in social norms, acceptance of IPA within relationships and/or sexual assault in general is also higher [56,68,69]. This link has been found in a variety of cultures around the world, including the Basque region of Spain [70], Latin America [71], Pakistan [72], Yugoslavia [73], Lebanon [74], Norway [75], South Asia [76], and East Asia [77,78,79], among others. While the individual culture of our participants was not measured, they all came from a rural part of the country (specifically, Indiana) that has a blend of Midwestern and Southern values. This part of the U.S. traditionally votes for conservative politicians and promotes old-fashioned values. These cultural influences may have affected participants’ views of what they believe is “normal” or “acceptable”, just as people in other countries are affected by their cultures or sub-cultures.

Even what “counts” as abuse changes within a culture over time. Some people perceive that “domestic violence” only refers to physical acts, such as punching or slapping, but that psychological aggression, such as insults, does not apply [80,81]. Too often, people react to hearing about cases of psychological and emotional abuse by stating, “At least they weren’t hit”. What is surprising about such statements is that they overlook how it can be more difficult to overcome an extended period of being belittled and humiliated than the occasional physical abuse [7]. The link between psychological abuse and feeling depressed or anxious is robust and occurs beyond instances of physical abuse [6,8,60]. Many people in the U.S. believe that perpetrators of physical violence should be sanctioned or punished, but that psychological aggression should not [82].

One woman we interviewed appeared to struggle with what threshold of IPA was acceptable, and she recognized that her perceptions were influenced by her family of origin and cultural expectations for gendered behavior. She stated, “Growing up, my parents physically fought all the time. My husband had seen his mother kill herself in the back yard when he was little, and his father died a few years later, so I understood where his temper came from.”

Finally, as discussed earlier, some victims of IPA may question what “counts” as violence and whether their partner’s behavior has met a cutoff point for what should be tolerated. As the aggression escalates, their standards may shift to higher acceptance as their commitment and investment increases. A particularly poignant example of this came from this final interview quotation, which exemplifies many of the processes described above, including self-blame, justification for her partner’s “temper”, hoping that things work out (“for better or worse”), and a specific avoidance of labeling his behavior as abusive because it did not include hitting her with his fists. This final quotation, combining various levels of influence discussed, demonstrates that the levels are not mutually exclusive. Rather, multiple influences often weave together to form a web of subjective perceptions that keep victims of IPA in their relationships:

“The only thing he’s really done is pushed. He threw a stand at me… but it didn’t hit me. He knows if he hits me, he’ll get his gun permit taken away. So he’s never really used his hands on me. He hit my daughter. He’s thrown several things at me. He’s kicked me. But that’s it… [Someone] broke [the furniture]. And it made him mad. That’s when he threw [the furniture] at me and punched the walls…. I’ve been lucky with him not hitting me. It’s mainly been kicking or shoving. I thought things would work out. Because sometimes people do have a temper, but then things do work out.”

## 3. Conclusions

We propose that there are multiple levels of social and contextual influence on how and why victims of IPA perceive and cognitively reframe their partner’s aggressive actions. We discussed four levels that span more immediate and personal influence to more global and distal. The more immediate contexts (e.g., individual factors) may influence people more because they are always relevant to the specific person in the center. Their motivation to minimize or reinterpret aggression may be about personal, concrete, immediate cognitive coping and self-protection—but those motivations will ultimately also be at least somewhat affected by their theoretical, abstract, or global views as well.

When victims do discount the aggression aimed toward them, it is not a harmless experience. Past researchers have noted that the tendency to downplay IPA results in “invisible harm” [5]. IPA “sets into motion a self-reinforcing cycle of depending on a partner, being brought down by a partner’s abuse, and feeling less strong and more dependent” ([5], p. 371). Moreover, despite IPA being the cause of personal distress, victims sometimes fail to see that their partner is the source of that distress [60]. This denial of their own unhappiness can lead victims to misunderstand their own situation and to stay in an abusive relationship after convincing themselves that they would be even more unhappy if that relationship ended [83].

A better understanding of how and why victims of IPA perceive what is happening to them is not an avenue to blaming them for biased perceptions or for staying in their relationships. Nor should the conclusion be that people stay in aggressive relationships because of sadist tendencies (see [84] for a discussion of reasons for staying). In fact, when partners rely on each other and are not emotionally (or financially) prepared to end their relationship, the rational decision is to stay with each other. 

Future research could deepen an understanding IPA in two ways. First, among the multiple levels of influence on victims’ cognitive reframing, one that needs to be examined further concerns the influence of *perpetrators*’ actions, manipulations, and efforts to keep victims dependent. Within couples, perpetrator efforts to bias attributions and cognitive processes are likely to be correlated with victims’ cognitive reframing, if both couple members are telling themselves the same story or if one is influencing the other. Other theories, such as interdependence theory, would shed light on dyadic patterns that predict cognitive reframing, as well as perpetrator behaviors that directly contribute to a victim’s dependence [84].

A second line of future research is also the creation of quantitative measures and analyses that would provide further support of the ideas suggested here. Such quantitative data could then be compared against the current qualitative interviews to determine whether the ideas and themes found here replicate across samples and measures. It is important to note that the specific factors identified in the current study are unlikely to be comprehensive; additional factors are very likely to be added by other scholars. Finally, quantitative measures might show changes in reframing and cognitive processes over time.

Interventions may be designed with the goal of instilling objective perceptions and assessing when and how relationship dynamics might change. Such interventions should be designed with each partner’s current mindset as a starting point, including their subjective commitment. Programs at shelters, police conversations with victims, policy makers, and members of a person’s social support network should consider these multiple levels of influence to create a complete picture of the emotional and psychological challenges that confront victims of IPA. The situation is never simple in the complicated dynamics of intimate partner aggression, but greater clarity and precise action may occur when the multiple factors at work are understood and addressed.

## References

[1] Deutsch M., Gerard H.B. (1955). A Study of Normative and Informational Social Influences upon Individual Judgment. J. Abnorm. Soc. Psychol..

[2] Rose S., Frieze I.H. (1993). Young singles contemporary dating scripts. Sex Roles.

[3] Simon W., Gagnon J.H. (1986). Sexual scripts—Permanence and change. Arch. Sex. Behav..

[4] Straus M.A. (1999). The controversy over domestic violence by women—A methodological, theoretical, and sociology of science analysis. Violence Intimate Relationships.

[5] Arriaga X.B., Cobb R.J., Daly C.A., Vangelisti A., Perlman D. (2018). Aggression and violence in romantic relationships. The Cambridge Handbook of Personal Relationships.

[6] Arias I., Pape K.T. (1999). Psychological Abuse: Implications for Adjustment and Commitment to Leave Violent Partners. Violence Vict..

[7] Follingstad D.R., Rutledge L.L., Berg B.J., Hause E.S., Polek D.S. (1990). The Role of Emotional Abuse in Physically Abusive Relationships. J. Fam. Violence.

[8] Lawrence E., Yoon J., Langer A., Ro E. (2009). Is Psychological Aggression as Detrimental as Physical Aggression? The Independent Effects of Psychological Aggression on Depression and Anxiety Symptoms. Violence Vict..

[9] Arriaga X.B., Capezza N.M., Shaver P.R., Mikulincer M. (2011). The Paradox of Partner Aggression: Being Committed to an Aggressive Partner. Human Aggression and Violence: Causes, Manifestations, and Consequences.

[10] Festinger L., Carlsmith J.M. (1959). Cognitive Consequences of Forced Compliance. J. Abnorm. Soc. Psychol..

[11] Fletcher G.J.O., Fitness J., Blampied N.M. (1990). The Link between Attributions and Happiness in Close Relationships: The Roles of Depression and Explanatory Style. J. Soc. Clin. Psychol..

[12] Kearns J.N., Fincham F.D. (2005). Victim and Perpetrator Accounts of Interpersonal Transgressions: Self-Serving or Relationship-Serving Biases?. Pers. Soc. Psychol. B.

[13] Arriaga X.B. (2002). Joking Violence Among Highly Committed Individuals. J. Interpers. Violence.

[14] Bronfenbrenner U. (1977). Toward an Experimental Ecology of Human-Development. Am. Psychol..

[15] Bronfenbrenner U., Ceci S.J. (1994). Nature-Nurture Reconceptualized in Developmental Perspective—A Bioecological Model. Psychol. Rev..

[16] Bronfenbrenner U. (2005). Making Human Beings Human: Bioecological Perspectives on Human Development.

[17] Bronfenbrenner U., Evans G.W. (2000). Developmental Science in the 21st Century: Emerging Questions, Theoretical Models, Research Designs and Empirical Findings. Soc. Dev..

[18] Bronfenbrenner U. (1994). Ecological Models of Human Development. Inter. Encycl. Educ..

[19] Garbarino J. (1976). Preliminary Study of Some Ecological Correlates of Child-Abuse—Impact of Socioeconomic Stress on Mothers. Child Dev..

[20] Cicchetti D., Lynch M. (1993). Toward an Ecological Transactional Model of Community Violence and Child Maltreatment—Consequences for Childrens Development. Psychiatry.

[21] Small S.A., Luster T. (1994). Adolescent Sexual-Activity—An Ecological, Risk-Factor Approach. J. Marriage Fam..

[22] Richard L., Gauvin L., Raine K. (2011). Ecological Models Revisited: Their Uses and Evolution in Health Promotion Over Two Decades. Annu. Rev. Publiv Health.

[23] Banyard V.L. (2011). Who Will Help Prevent Sexual Violence: Creating an Ecological Model of Bystander Intervention. Psychol. Violence.

[24] Nelson J.R., Lund E.M., Johnson A.J., Nelson J.R., Lund E.M. (2017). Bronfenbrenner’s theoretical framework adapted to women with disabilities experiencing intimate partner violence. Religion, Disability, and Interpersonal Violence.

[25] Nurius P.S., Norris J. (1995). A cognitive ecological model of women’s response to male sexual coercion in dating. J. Psychol. Hum. Sex..

[26] Slep A.M.S., Foran H.M., Heyman R.E., Snarr J.D. (2010). Unique Risk and Protective Factors for Partner Aggression in a Large Scale Air Force Survey. J. Commun. Health.

[27] Campbell R., Dworkin E., Cabral G. (2009). An Ecological Model of the Impact of Sexual Assault on Women’s Mental Health. Trauma Violence Abus..

[28] Black D.S., Sussman S., Unger J.B. (2010). A Further Look at the Intergenerational Transmission of Violence: Witnessing Interparental Violence in Emerging Adulthood. J. Interpers. Violence.

[29] Ehrensaft M.K., Cohen P., Brown J., Smailes E., Chen H.N., Johnson J.G. (2003). Intergenerational Transmission of Partner Violence: A 20-Year Prospective Study. J. Consult. Clin. Psychol..

[30] Arriaga X.B., Capezza N.M., Daly C.A. (2016). Personal Standards for Judging Aggression by a Relationship Partner: How Much Aggression is Too Much?. J. Pers. Soc. Psychol..

[31] Dutton D.G., Bodnarchuk M., Kropp R., Hart S.D., Ogloff J.P. (1997). Client Personality Disorders Affecting Wife Assault Post-Treatment Recidivism. Violence Vict..

[32] Holtzworth-Munroe A., Meehan J.C., Herron K., Rehman U., Stuart G.L. (2000). Testing the Holtzworth-Munroe and Stuart (1994) batterer typology. J. Consult. Clin. Psychol..

[33] Jacobsen N., Gottman J. (1998). When Men Batter Women: New Insights into Ending Abusive Relationships.

[34] Holtzworthmunroe A., Stuart G.L. (1994). Typologies of Male Batterers—3 Subtypes and the Differences among Them. Psychol. Bull..

[35] Bell K.M., Orcutt H.K. (2009). Posttraumatic Stress Disorder and Male-Perpetrated Intimate Partner Violence. JAMA J. Am. Med. Assoc..

[36] Kirby A.C., Beckham J.C., Calhoun P.S., Roberts S.T., Taft C.T., Elbogen E.B., Dennis M.F. (2012). An Examination of General Aggression and Intimate Partner Violence in Women with Posttraumatic Stress Disorder. Violence Vict..

[37] Semiatin J.N., Torres S., LaMotte A.D., Portnoy G.A., Murphy C.M. (2017). Trauma Exposure, PTSD Symptoms, and Presenting Clinical Problems Among Male Perpetrators of Intimate Partner Violence. Psychol. Violence.

[38] Taft C.T., Schumm J., Orazem R.J., Meis L., Pinto L.A. (2010). Examining the Link Between Posttraumatic Stress Disorder Symptoms and Dating Aggression Perpetration. Violence Vict..

[39] Buchholz K.R., Bohnert K.M., Sripada R.K., Rauch S.A.M., Epstein-Ngo Q.M., Chermack S.T. (2017). Associations Between PTSD and Intimate Partner and Non-Partner Aggression among Substance Using Veterans in Specialty Mental Health. Addict. Behav..

[40] Creech S.K., Macdonald A., Benzer J.K., Poole G.M., Murphy C.M., Taft C.T. (2017). PTSD Symptoms Predict Outcome in Trauma-Informed Treatment of Intimate Partner Aggression. J. Consult. Clin. Psychol..

[41] Taft C.T., Street A.E., Marshall A.D., Dowdall D.J., Riggs D.S. (2007). Posttraumatic stress disorder, anger, and partner abuse among Vietnam combat veterans. J. Fam. Psychol..

[42] Taft C.T., Weatherill R.P., Woodward H.E., Pinto L.A., Watkins L.E., Miller M.W., Dekel R. (2009). Intimate Partner and General Aggression Perpetration Among Combat Veterans Presenting to a Posttraumatic Stress Disorder Clinic. Am. J. Orthopsychiat..

[43] Teten A.L., Schumacher J.A., Taft C.T., Stanley M.A., Kent T.A., Bailey S.D., Dunn N.J., White D.L. (2010). Intimate Partner Aggression Perpetrated and Sustained by Male Afghanistan, Iraq, and Vietnam Veterans With and Without Posttraumatic Stress Disorder. J. Interpers. Violence.

[44] Sexton M.B., Davis A.K., Buchholz K.R., Winters J.J., Rauch S.A.M., Yzquibell M., Bonar E.E., Friday S., Chermack S.T. (2018). Veterans with recent substance use and aggression: PTSD, substance use, and social network behaviors. Psychol. Trauma-US.

[45] Weiss N.H., Duke A.A., Overstreet N.M., Swan S.C., Sullivan T.P. (2016). Intimate partner aggression-related shame and posttraumatic stress disorder symptoms: The moderating role of substance use problems. Aggress. Behav..

[46] Weiss N.H., Duke A.A., Sullivan T.P. (2014). Evidence for a curvilinear dose-response relationship between avoidance coping and drug use problems among women who experience intimate partner violence. Anxiety Stress Coping.

[47] Cunradi C.B., Ames G.M., Duke M. (2011). The Relationship of Alcohol Problems to the Risk for Unidirectional and Bidirectional Intimate Partner Violence among a Sample of Blue-Collar Couples. Violence Vict..

[48] Eckhardt C.I. (2007). Effects of alcohol intoxication on anger experience and expression among partner assaultive men. J. Consult. Clin. Psychol..

[49] Fals-Stewart W. (2003). The occurrence of partner physical aggression on days of alcohol consumption: A longitudinal diary study. J. Consult. Clin. Psychol..

[50] Moore T.M., Elkins S.R., McNulty J.K., Kivisto A.J., Handsel V.A. (2011). Alcohol Use and Intimate Partner Violence Perpetration Among College Students: Assessing the Temporal Association Using Electronic Diary Technology. Psychol. Violence.

[51] Smith P.H., Homish G.G., Leonard K.E., Cornelius J.R. (2012). Intimate Partner Violence and Specific Substance Use Disorders: Findings from the National Epidemiologic Survey on Alcohol and Related Conditions. Psychol. Addict. Behav..

[52] Witte T.H., Kopkin M.R., Hollis S.D. (2015). Is It Dating Violence or Just “Drunken Behavior”? Judgments of Intimate Partner Violence When the Perpetrator Is Under the Influence of Alcohol. Subst. Use Misuse.

[53] Neal A.M., Edwards K.M. (2017). Perpetrators’ and Victims’ Attributions for IPV: A Critical Review of the Literature. Trauma Violence Abus..

[54] Capaldi D.M., Shortt J.W., Kim H.K., Pinsof W.M., Lebow J.L. (2005). A life span developmental systems perspective on aggression toward a partner. Family Psychology: The Art of the Science.

[55] Capaldi D.M., Kim H.K. (2007). Typological Approaches to Violence in Couples: A Critique and Alternative Conceptual Approach. Clin. Psychol. Rev..

[56] Johnson M.P. (1995). Patriarchal Terrorism and Common Couple Violence—2 Forms of Violence against Women. J. Marriage Fam..

[57] Johnson M.P., O’Toole L.L., Schiffman J.R., Edwards M.L.K. (2007). Domestic violence: The intersection of gender and control. Gender Violence: Interdisciplinary Perspectives.

[58] Finkel E.J. (2007). Impelling and inhibiting forces in the perpetration of intimate partner violence. Rev. Gen. Psychol..

[59] Tan K., Arriaga X.B., Agnew C.R. (2018). Running on empty: Measuring psychological dependence in close relationships lacking satisfaction. J. Soc. Pers. Relat..

[60] Arriaga X.B., Schkeryantz E.L. (2015). Intimate Relationships and Personal Distress: The Invisible Harm of Psychological Aggression. Pers. Soc. Psychol. Bull..

[61] Mills R.B., Malley-Morrison K. (1998). Emotional commitment, normative acceptability, and attributions for abusive partner behaviors. J. Interpers. Violence.

[62] Katz J., Arias I., Beach S.R.H., Brody G., Roman P. (1995). Excuses, excuses: Accounting for the effects of partner violence on marital satisfaction and stability. Violence Vict..

[63] Gordon K.C., Burton S., Porter L. (2004). Predicting the intentions of women in domestic violence shelters to return to partners: Does forgiveness play a role?. J. Fam. Psychol..

[64] Rusbult C.E., Agnew C.R., Arriaga X.B., Van Lange P.A.M., Kruglanski A.W., Higgins E.T. (2012). The investment model of commitment processes. Handbook of Theories of Social Psychology; Volume 2.

[65] Goodfriend W., Agnew C.R. (2008). Sunken Costs and Desired Plans: Examining Different Types of Investments in Close Relationships. Pers. Soc. Psychol. B.

[66] Rosen K.H., Cahn D.D., Lloyd S.A. (1996). The ties that bind women to violent premarital relationships: Processes of seduction and entrapment. Family Violence from a Communication Perspective.

[67] Gelles R., Loseke R.J.G.D.R. (1993). Through a sociological lens: Social structure and family violence. Current Controversies on Family Violence.

[68] Dobash R.E., Dobash R. (1979). Violence against Wives: A Case Against the Patriarchy.

[69] Garcia-Moreno C., Jansen H.A.F.M., Ellsberg M., Heise L., Watts C.H. (2006). Prevalence of intimate partner violence: Findings from the WHO multi-country study on women’s health and domestic violence. Lancet.

[70] Ibabe I., Arnoso A., Elgorriaga E. (2016). Ambivalent Sexism Inventory: Adaptation to Basque Population and Sexism as a Risk Factor of Dating Violence. Span. J. Psychol..

[71] Rondon M.B. (2003). From Marianism to terrorism: The many faces of violence against women in Latin America. Arch. Women Ment. Health.

[72] Zakar R., Zakar M.Z., Kraemer A. (2013). Men’s Beliefs and Attitudes toward Intimate Partner Violence against Women in Pakistan. Violence Against Women.

[73] Valentich M. (1994). Rape revisited: Sexual violence against women in the former Yugoslavia. Can. J. Hum. Sex..

[74] Obeid N., Chang D.F., Ginges J. (2010). Beliefs about Wife Beating: An Exploratory Study with Lebanese Students. Violence Against Women.

[75] Bendixen M., Henriksen M., Nostdahl R.K. (2014). Attitudes toward Rape and Attribution of Responsibility to Rape Victims in A Norwegian Community Sample. Nord. Psychol..

[76] Chowdhury E.H. (2015). Rethinking Patriarchy, Culture and Masculinity: Transnational Narratives of Gender Violence and Human Rights Advocacy. J. Int. Women Stud..

[77] Kim J.Y., Emery C. (2003). Marital power, conflict, norm consensus, and marital violence in a nationally representative sample of Korean couples. J. Interpers. Violence.

[78] Ozaki R., Otis M.D. (2017). Gender Equality, Patriarchal Cultural Norms, and Perpetration of Intimate Partner Violence: Comparison of Male University Students in Asian and European Cultural Contexts. Violence Against Women.

[79] Yoshihama M. (2005). A web in the patriarchal clan system—Tactics of intimate partners in the Japanese sociocultural context. Violence Against Women.

[80] Capezza N.M., Arriaga X.B. (2008). You Can Degrade but You Can’t Hit: Differences in Perceptions of Psychological Versus Physical Aggression. J. Soc. Pers. Relat..

[81] Carlson B.E., Worden A.P. (2005). Attitudes and beliefs about domestic violence: Results of a public opinion survey—I. Definitions of domestic violence, criminal domestic violence, and prevalence. J. Interpers. Violence.

[82] Sorenson S.B., Taylor C.A. (2005). Female aggression toward male intimate partners: An examination of social norms in a community-based sample. Psychol. Women Quart..

[83] Arriaga X.B., Capezza N.M., Goodfriend W., Rayl E.S., Sands K.J. (2013). Individual Well-Being and Relationship Maintenance at Odds: The Unexpected Perils of Maintaining a Relationship With an Aggressive Partner. Soc. Psychol. Pers. Sci..

[84] Rusbult C.E., Martz J.M. (1995). Remaining in an Abusive Relationship—An Investment Model Analysis of Nonvoluntary Dependence. Pers. Soc. Psychol. B.

